# A Hybrid Method for Image Segmentation Based on Artificial Fish Swarm Algorithm and Fuzzy *c*-Means Clustering

**DOI:** 10.1155/2015/120495

**Published:** 2015-11-16

**Authors:** Li Ma, Yang Li, Suohai Fan, Runzhu Fan

**Affiliations:** ^1^School of Information Science and Technology, Jinan University, Guangzhou 510632, China; ^2^International School, Jinan University, Guangzhou 510632, China

## Abstract

Image segmentation plays an important role in medical image processing. Fuzzy *c*-means (FCM) clustering is one of the popular clustering algorithms for medical image segmentation. However, FCM has the problems of depending on initial clustering centers, falling into local optimal solution easily, and sensitivity to noise disturbance. To solve these problems, this paper proposes a hybrid artificial fish swarm algorithm (HAFSA). The proposed algorithm combines artificial fish swarm algorithm (AFSA) with FCM whose advantages of global optimization searching and parallel computing ability of AFSA are utilized to find a superior result. Meanwhile, Metropolis criterion and noise reduction mechanism are introduced to AFSA for enhancing the convergence rate and antinoise ability. The artificial grid graph and Magnetic Resonance Imaging (MRI) are used in the experiments, and the experimental results show that the proposed algorithm has stronger antinoise ability and higher precision. A number of evaluation indicators also demonstrate that the effect of HAFSA is more excellent than FCM and suppressed FCM (SFCM).

## 1. Introduction

Image segmentation is to divide the image into regions with different features. As an important procedure in image processing, image segmentation is a hotspot and difficulty in medical image technology field [1]. One of the most widely used algorithms in the area of image segmentation [2, 3] is the fuzzy *c*-means (FCM). FCM [4–6] is the mainstream algorithm in fuzzy clustering method. It has advantages of unsupervised, simple implementation, no threshold set, and practicality, but at the same time it has the disadvantages of sensitivity to random initial value, easily falling into local optimal solution, and large calculation under the multidimensional space. Intelligent algorithm can obtain global optimal solution quickly, and it is suitable for the complex data space of nonlinear multidimension while few approaches aim at combining AFSA with FCM. As early as 1996, Chun and Yang have introduced intelligent algorithm into image segmentation field by researching the combination of genetic algorithm and FCM [7]. The hybrid algorithms [8–12] have more excellent performance to overcome the shortcomings of FCM.

Artificial fish swarm algorithm (AFSA) [13, 14] is an algorithm which is put forward by Li et al. It has features of universality, combining with the traditional algorithm easily, and less sensitivity to initial value, and its theory is suitable for solving the clustering problem. For years, some scholars introduced AFSA into the field of image segmentation [15–18]. Most of the approaches aim at the optimization of threshold segmentation method, while the approach on combining AFSA with FCM is less. Wang et al. [19] utilized AFSA to get the optimal clustering centers and then carried out the local searching by FCM so as to avoid local optimum. Liu et al. [20] proposed a dynamic fuzzy clustering method based on artificial fish swarm algorithm by introducing a fuzzy equivalence matrix. He et al. [21] verified that AFSA with adaptive visual and step combining with FCM is more superior to genetic algorithm.

In this paper, we propose a hybrid artificial fish swarm clustering algorithm (HAFSA). The proposed algorithm combines artificial fish swarm algorithm (AFSA) with FCM. With parallel search technology, HAFSA can overcome the defect that the FCM is easy to fall into local optimal solution. Metropolis criterion and noise reduction mechanism are introduced into the proposed algorithm, not only improving the convergence rate but also taking advantage of spatial neighborhood information to enhance antinoise ability and practicability. The artificial grid graph and Magnetic Resonance imaging (MRI) are utilized in the experiments. The experimental results compared with FCM and SFCM [22] show that HAFSA is more excellent through a number of evaluation indicators. These verify that HAFSA is effective and feasible in overcoming the sensitivity of initial value and noise.

## 2. Fuzzy *c*-Means Algorithm

FCM algorithm was proposed by Dunn [5] and improved by Bezdek [6] later. The basic FCM algorithm can divide the gray image data into several clusters. The optimization model of FCM is shown in the following formula:

(1) min JU,V=∑i=1c ∑j=1nuijmdij2,s.t. ∑i=1cuij=1,∀j=1⋯n,

where **U** = {*u*_*ij*_}_*c*×*n*_ denotes the fuzzy membership matrix, *u*_*ij*_ ∈ [0,1] is the degree of membership of the pixel *x*_*j*_ in the cluster *v*_*i*_, and *n* = *M* × *N* is the size of gray image. **V** = {*v*_*i*_}_*c*_ denotes *c* cluster centers. *d*_*ij*_ = ‖*v*_*i*_ − *x*_*j*_‖ is the Euclidean distance between *x*_*j*_ and *v*_*i*_. The fuzzy weighting exponent *m* ∈ [1, *∞*) is generally set to 2. The update equations of membership degree matrix and cluster centers are shown in the following formula:

(2) uij=∑jk=1cdijdkj2/m−1−1,vi=∑j=1nuijmxj·∑j=1nuijm−1.

FCM algorithm has simple principle and process without initial parameters. However, FCM is sensitive to initial membership degree matrix (or cluster centers) and falls easily into local optimal solution because of the iterative process of gradient descent. Images are frequently attacked by noises. FCM is not suitable for the actual image without the ability to identify noise. At present, scholars improved the defects of FCM by changing the objective function or combining with other optimization algorithms [23, 24]. SFCM [22] was proposed to improve the clustering performance as well as convergence rate by introducing the suppression factor. Moreover, some researchers educed noise by spatial information [25, 26] or noise processing [27].

## 3. Hybrid Artificial Fish Swarm Algorithm (HAFSA)

### 3.1. Artificial Fish Swarm Algorithm (AFSA)

AFSA is an intelligent optimization algorithm which is designed by imitating the behaviors of fish swarm. The algorithm is an autonomous model based on four major behaviors, preying behavior, swarming behavior, following behavior, and random behavior, which fully perform the local search so that the population diversity is ensured maximally and local optimal solution avoids premature convergence.

**Figure 1:**
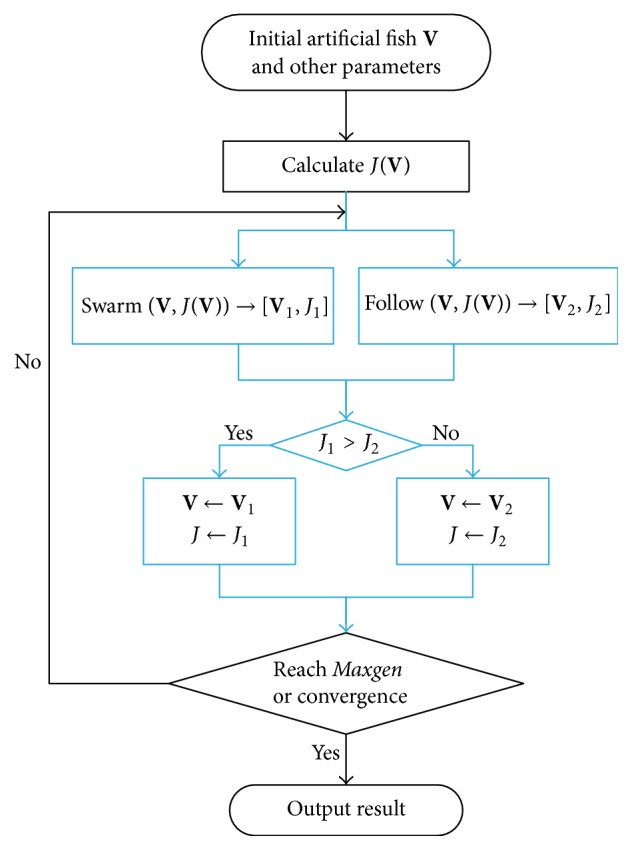
The flowchart of AFSA.

Let *G* = {0,1,…, *L* − 1} denote the gray levels, where *L* = 256 and gray levels are from 0 to 255. In the process of image segmentation, fish's location coding is a pixel gray value vector **V** = (*v*_1_, *v*_2_,…,*v*_*c*_)^*T*^ which consisted of *c* cluster centers. The *c*-means objective function serves as food concentration of artificial fish; that is, *F*(**V**) = *J*(**V**). As shown in Figure 1, AFSA mainly adjusts the position of the artificial fish by swarm behavior and follow behavior. AFSA will be terminated when it converges or achieves the maximum generation* Maxgen*. The four basic behaviors are described below.

*(i) Prey Behavior*. Prey behavior is the default behavior of the swarm behavior and follow behavior. Assuming the current position of artificial fish is **V**_*i*_, another position **V**_*j*_ is randomly selected in the visual field of **V**_*i*_. If *J*(**V**_*j*_) < *J*(**V**_*i*_), the artificial fish moves a step to **V**_*j*_ according to formula (3) and prey behavior terminates. If *J*(**V**_*j*_) ≥ *J*(**V**_*i*_), keep reselecting new **V**_*j*_ until *J*(**V**_*j*_) < *J*(**V**_*i*_) or reach the maximum reselection times *try*_*number*. Artificial fish will perform the random behavior if it still cannot select an ideal position after *try*_*number* times reselection. Consider

(3) $$\begin{array}{c} V_{i}⟵V_{i}+\xi \cdot step\cdot \frac{V_{j}-V_{i}}{\left\|V_{j}-V_{i}\right\|}, \end{array}$$

where *step* is the maximum moving distance of artificial fish and *ξ* is the random scalar.

*(ii) Swarm Behavior.* If *J*(**V**_*c*_)/num < *δ* · *J*(**V**_*i*_), **V**_*c*_ will have more food concentration and will be more uncrowded than **V**_*i*_; then the artificial fish should move a step toward **V**_*c*_ according to formula (3) (**V**_*j*_ ← **V**_*c*_); else perform the prey behavior, where integer num denotes the number of partners in the visual field of **V**_*i*_, **V**_*c*_ is the center position of num partners, and *δ* ∈ [0,1] is the congestion level.

*(iii) Follow Behavior.* If *J*(**V**_*j*_^*∗*^)/num < *δ* · *J*(**V**_*i*_), **V**_*j*_^*∗*^ will have more food concentration and will be more uncrowded than **V**_*i*_; then the artificial fish should move a step toward **V**_*j*_^*∗*^ according to formula (3) (**V**_*j*_ ← **V**_*j*_^*∗*^); else perform the prey behavior, where the partner position **V**_*j*_^*∗*^ has minimal *c*-means objective function *J* in visual field of **V**_*i*_.

*(iv) Random Behavior*. Random behavior is the default of prey behavior. The artificial fish in position **V**_*i*_ should perform the random behavior according to formula (4) when it cannot select a more excellent position in prey behavior after *try*_*number* times random reselection, where **η** denotes a random vector in [−1,1] with the size of *c* × 1. Consider

(4) $$\begin{array}{c} V_{i}⟵V_{i}+visual\cdot \eta . \end{array}$$

### 3.2. Metropolis Criterion

Metropolis criterion stems from simulated annealing algorithm (SA) [28]. In HAFSA, Metropolis criterion exists in each iteration behind the swarm behavior and follow behavior. Cluster center vector **V**_*i*_ and the *c*-means objective function *J*(**V**_*i*_) are equivalent to the state of the atom and the energy function in SA. After swarm behavior and follow behavior, the artificial fish located in the original position **V**_*i*_ will move a step to a new position **V**_*j*_. If *J*(**V**_*j*_) ≤ *J*(**V**_*i*_), the artificial fish should accept superior position **V**_*j*_ unconditionally. If *J*(**V**_*j*_) > *J*(**V**_*i*_), the artificial fish should accept inferior position by probability *P*, which is defined as formula (5), or refuse **V**_*j*_ and turn back to **V**_*i*_. Consider

(5) $$\begin{array}{c} P=\exp\left(\;\frac{J\left(V_{j}\right)-J\left(V_{i}\right)}{k\cdot T_{t}}\;\right), \end{array}$$

where *k* = 1 is Boltzmann constant, *T*_*t*_ = *T*_*t*−1_ · *q* is the temperature of the *t*th iteration, *q* = (*T*_end_/*T*_0_)^1/*Maxgen*^ is the refrigeration level, *T*_0_ is the initial temperature, and *T*_end_ is the final temperature.

In HAFSA, Metropolis criterion is utilized to distinguish whether the artificial fish should accept a new position. At the beginning of HAFSA, the probability of accepting inferior position is larger, the diversity of population is increased, and the wide area searching is enlarged to avoid falling into local optimum. With the increasing of iterations, the acceptance probability decreases and more and more inferior position will be refused; then the convergence speed slows down but the local search ability will be enlarged which is helpful for keeping global optimal position.

### 3.3. Noise Reduction Mechanism

**Figure 2:**
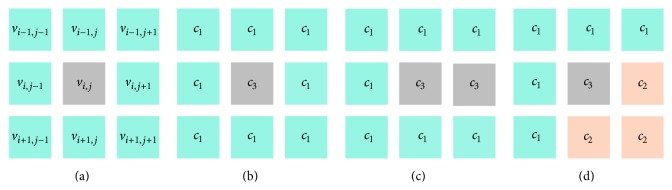
Noise pixel and neighbor regions.

Most noises are Gaussian noise and salt and pepper noise in medical images. Noises generally locate in the same cluster or boundary of several different clusters. The gray image is mostly like a grid network where each pixel possesses 8 neighbors (except the pixels at the edge of the image) as shown in Figure 2(a). Different from the common pixels, the noise pixels usually have huge disparities with most of their neighbors. In previous articles, the noises usually are processed by filter, but the filter is only suitable for specific images which will lead to the deterioration of segmentation accuracy for edge blur. A noise reduction mechanism is proposed in this section which integrates into AFSA and achieves the identification and procession of noises. Gaussian noise is chosen to test the antinoise ability of HAFSA.

In AFSA, artificial fish will obtain the best cluster center **V**_best_ with minimal *c*-means objective function *J* after each iterative computation. According to **V**_best_, we can calculate the fuzzy membership matrix **U** and then the best cluster matrix **C** by the value of the *c*-means objective function. Consider

(6) $$\begin{array}{c} C=\left[\begin{array}{cccc} c_{1,1} & c_{1,2} & \cdots & c_{1,n}\\ c_{2,1} & c_{2,2} & \cdots & c_{2,n}\\ \vdots & \vdots & \ddots & \vdots \\ c_{m,1} & c_{m,2} & \cdots & c_{m,n} \end{array}\right], \end{array}$$

where *c*_*i*,*j*_ ∈ {1,…, *c*} denotes the cluster attribute of pixel *v*_*i*,*j*_. Let 8 nearest pixels be the neighbors of *v*_*i*,*j*_. When the number of neighbors cluster attributes which are equal to *c*_*i*,*j*_ is less than *k*, *v*_*i*,*j*_ can be defined as a noise pixel, where *k* is an arbitrary integer and mostly set as 1. At the end of each iteration, after recognizing all the noises, we replace noise pixel with an average pixel of the neighbors which belong to the maximum cluster set. With the convergence of AFSA, the noise in the gray image will be recognized and reduced step by step.

As an example, Figure 2(a) demonstrates that the pixel of candidate noise *v*_*i*,*j*_ generally has a huge difference with its 8 neighbors.  Figure 2(b) demonstrates that *v*_*i*,*j*_ has been signed as cluster attributes *c*_3_ by AFSA, but its neighbors have been signed as cluster attributes *c*_1_. There are no neighbors signed as *c*_3_, and then *v*_*i*,*j*_ can be recognized as a noise. As the density of noise increases, we can set larger *k* to recognize adjacent noises. As shown in Figure 2(c), the adjacent noises *v*_*i*,*j*_ and *v*_*i*,*j*+1_ will be recognized if *k* = 2. After recognizing noises, we will replace noise with average pixel of its neighbors.  Figure 2(d) demonstrates a complex circumstance, where the neighbors of noise have been divided into 2 different clusters *c*_1_ and *c*_2_; means noise *v*_*i*,*j*_ locates in the boundary of 2 clusters. The size of cluster *c*_1_ is 5, and the size of *c*_3_ is 3; hence we replace *v*_*i*,*j*_ with the average pixel of 5 neighbors in *c*_1_ as *v*_*i*,*j*_ ← (*v*_*i*−1,*j*−1_ + *v*_*i*−1,*j*_ + *v*_*i*−1,*j*+1_ + *v*_*i*,*j*−1_ + *v*_*i*+1,*j*−1_)/5.

An effective integration is designed by combining the noise reduction mechanism with AFSA, which processes the noise based on the best cluster center after each iterative concentration and reduces the calculation. Noise reduction mechanism always exists in the iterative process of AFSA and then subsequently converges. The procession of noise reduction gradually avoids the misjudgment of noise and ensures the accuracy and adaptability of noise identification.

### 3.4. Hybrid Artificial Fish Swarm Algorithm (HAFSA)

HAFSA is proposed in this section by introducing Metropolis criterion and noise reduction mechanism, based on AFSA. HAFSA inherits the computing framework of AFSA which has the advantages of strong optimization ability and less easiness of falling into the local optimum. By introducing Metropolis criterion, HAFSA's convergence speed has been accelerated and segmentation accuracy has been improved. The proposed noise reduction mechanism makes full use of AFSA's cluster result, which can improve the noise identification ability of HAFSA; meanwhile it has fewer computations than other noise reduction mechanisms. HAFSA is shown in Algorithm 1.

## 4. Experimental Results

The experiments are based on the computer with Intel Core i3, 4GB RAM, and dual-core 3.40 MHz and were performed in Matlab 2009a compiler. In order to verify FCM, SFCM, and HAFSA, artificial grid graph and Magnetic Resonance Imaging (MRI) (mr030.pgm in http://decsai.ugr.es/cvg/dbimagenes/gbio256.php) have been introduced to test the antinoise ability and the segmentation effect on real-world environment. In addition to the visually qualitative results, we have introduced many numerical indexes to evaluate the accuracy of segmentation results.

In HAFSA, we set the maximum number of iterations as *Maxgen* = 50, fuzzy weighting exponent as *m* = 2, food concentration as formula (3), the size of population as 20, visual field as *visual* = *L*/10, congestion level as *try*_*num* = 3, step of self-adaption as *step* = *visual*/2, initial temperature as *T*_0_ = 10^4^, and final temperature as *T*_end_ = 10^2^. Iterative threshold in FCM and SFCM is 10*e* − 5.

### 4.1. Assessment of Segmentation Performance

In this section, numerous evaluation indexes [24] have been carried out to evaluate the segmentation of HAFSA, FCM, and SFCM. They are *c*-means objective function value *J*, Peak Signal-to-Noise Ratio (PSNR), mean square error (MSE), variance partition coefficient (vpc), variance partition entropy (vpe), segmentation accuracy (Accuracy), and Jaccard similarity (JS). With the increasing of *J* and vpe and the decreasing of PSNR, vpc, Accuracy, and JS, the algorithms' segmentation results will get better. The calculation of evaluation indexes is described below.

PSNR is an objective standard to evaluate images which calculates the mean square error between original image and processed image whose computational formula is shown in formula (7). MSE is the mean square error between original image and processed image whose formula is shown in formula (8). Consider the following:

(7) PSNR=10·lg⁡xmax2·M·N∑i=1M∑j=1Nxi,j−yi,j2,

(8) MSE=1M·N·∑i=1M ∑j=1Nxi,j−yi,j2,

where *x*(*i*, *j*) is the gray level of the pixel in original image, *y*(*i*, *j*) is the gray level of the pixel in processed image, and *x*_max_ is the maximum gray level of the image.

Partition coefficient, vpc, and partition entropy, vpe, are defined in the following formula:

(9) vpc=1n·∑i=1c ∑j=1Nuij2,vpe=−1n·∑i=1c ∑j=1Nuijlog⁡uij.

Segmentation accuracy is defined as the sum of the correctly classified pixels divided by the sum of the total number of pixels; the computational formula is shown in the following formula:

(10) $$\begin{array}{c} Accuracy=\overset{c}{\underset{j=1}{\sum }}\frac{S_{1i}\cap S_{2i}}{\sum_{j=1}^{c}S_{2j}}, \end{array}$$

where *S*_1*i*_ represents the set of pixels belonging to the *i*th cluster by the segmentation algorithm and *S*_2*i*_ represents the set of pixels belonging to the *i*th cluster in the reference segmented image.

Jaccard similarity (JS) is applied as the metric to quantitatively evaluate the segmentation accuracy. JS is defined as follows:

(11) $$\begin{array}{c} JS\left(\;S_{1},S_{2}\;\right)=\frac{\left|S_{1}\cap S_{2}\right|}{\left|S_{1}\right|+\left|S_{2}\right|}, \end{array}$$

where *S*_1_ and *S*_2_ represent segmentation results of different algorithms and ground truth, respectively.

### 4.2. Segmentation of Artificial Grid Graph

**Figure 3:**
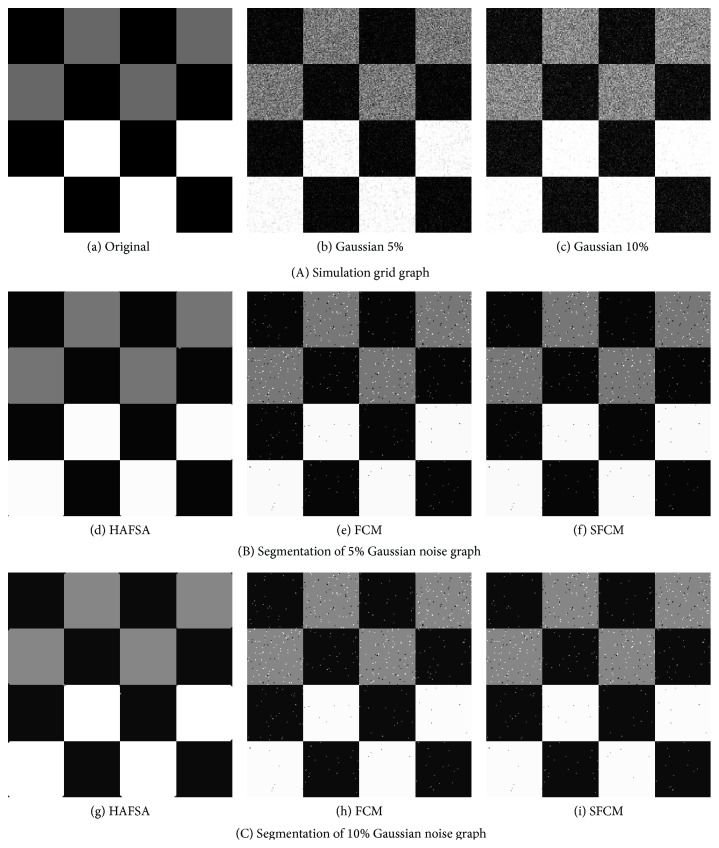
Segmentation of artificial grid graph. (a) An artificial grid graph. (b) Graph with 5% Gaussian noise. (c) Graph with 10% Gaussian noise. (d) HAFSA on the graph with 5% Gaussian noise. (e) FCM on the graph with 5% Gaussian noise. (f) SFCM on the graph with 5% Gaussian noise. (g) HAFSA on the graph with 10% Gaussian noise. (h) FCM on the graph with 10% Gaussian noise. (i) SFCM on the graph with 10% Gaussian noise.

In this section, we design the artificial grid graph to test the antinoise ability of HAFSA, FCM, and SFCM.  Figure 3(a) demonstrates the artificial grid graph which consisted of 3 kinds of pixels: black (0), gray (127), and white pixels (255). The grid graph can be divided into 4 × 4, a total of 16, sublumps according to the pixel level. The artificial grid graph is an ideal simulation object which has clear boundaries between different lumps. In order to test antinoise ability, 5% Gaussian noise and 10% Gaussian noise have been added to the artificial grid graph and produced Figures 3(b) and 3(c). Figures 3(d), 3(e), and 3(f) are the segmentation results of HAFSA, FCM, and SFCM on 5% Gaussian noise grid graph. Figures 3(g), 3(h), and 3(i) are the segmentation results of HAFSA, FCM, and SFCM on 10% Gaussian noise grid graph.

As it can be seen in Figure 3, compared with FCM and SFCM, the segmentation results of HAFSA have clear boundaries and almost no noise pixel under the 5% and 10% Gaussian noises, which verifies the strong antinoise ability of HAFSA. Even though the segmentation results of SFCM are slightly superior to FCM, both of them are unable to identify or eliminate noise, and their segmentation results are seriously attacked by noises.

In order to verify the performances among these three algorithms, evaluation indexes have been introduced to make a comprehensive assessment. We take the average of 30 times experiment results for each algorithm.  Table 1 presents 7 evaluation indexes of 3 algorithms on 3 kinds of artificial grid graph. From Table 1, it can be seen that all the evaluation indexes of 3 algorithms on grid graph have reached their best value, which means that 3 algorithms can get excellent segmentation on the no-noise condition. The evaluation indexes of HAFSA are more superior to FCM and SFCM under 5% and 10% Gaussian noises. It demonstrates that HAFSA has outstanding antinoise ability.

**Figure 4:**
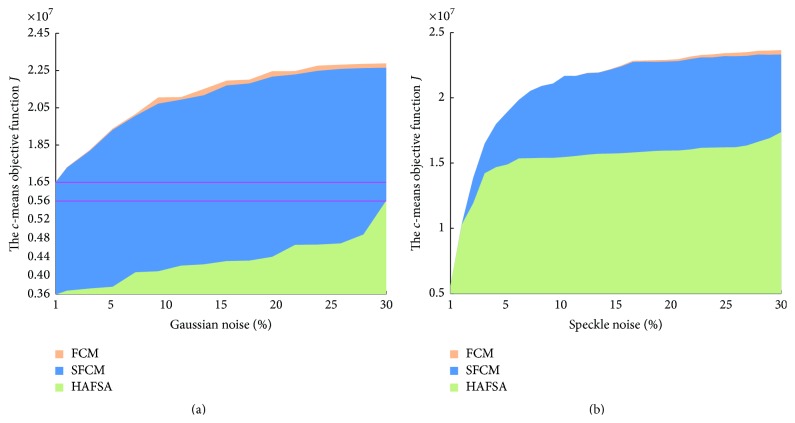
(a) The variation of the *c*-means objective function *J* on HAFSA, FCM, and SFCM with Gaussian noises. (b) The variation of the *c*-means objective function *J* on HAFSA, FCM, and SFCM with Speckle noises.

Figures 4(a) and 4(b) show curves of the relationship between *c*-means objective function *J* and the intensity of Gaussian noise and Speckle noise. According to Figures 4(a) and 4(b), the value *J* of 3 algorithms increases with the increasing intensity of Gaussian noise or Speckle noise. Under the same noise intensity situation, HAFSA is superior (with smaller *J*) to SFCM obviously, and SFCM algorithm is superior to FCM algorithm slightly. With the increasing of the Gaussian noise, *J* increases rapidly for SFCM and FCM but slowly for HAFSA (note that in Figure 4(a) *y*-axis is uneven; the spacing of *y*-axis corresponding by the lower red line is 0.2 times that of the upper red line). Figures 4(a) and 4(b) represent that HAFSA is an ideal image segmentation method with strong and robust antinoise ability.

### 4.3. Segmentation of MRI

**Figure 5:**
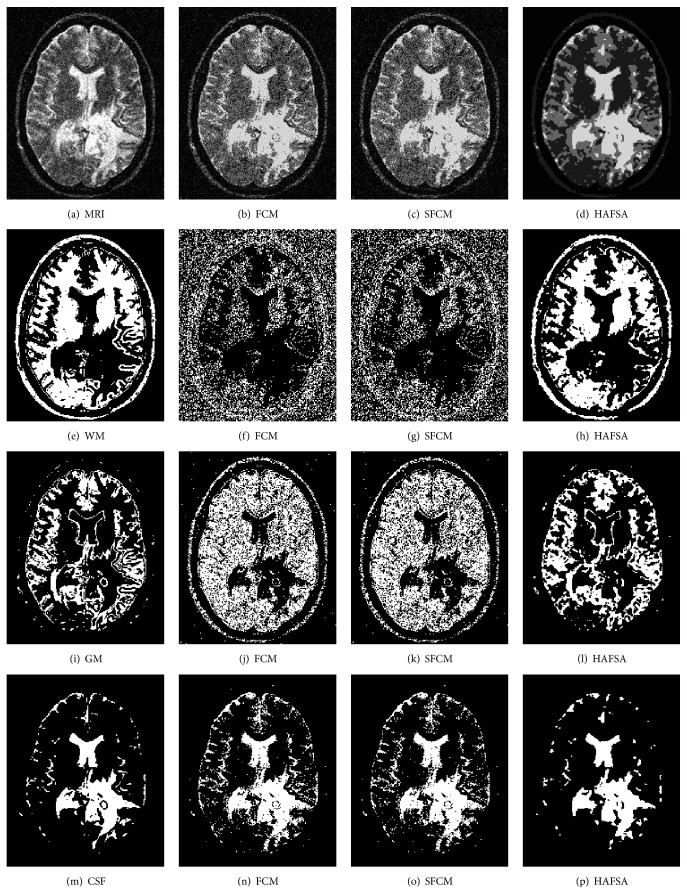
Segmentation results of MRI: (a) MRI; (b) FCM on MRI; (c) SFCM on MRI; (d) HAFSA on MRI; (e) WM (standard segmentation); (f) WM (FCM); (g) WM (SFCM); (h) WM (HAFSA); (i) GM (standard segmentation); (j) GM (FCM); (k) GM (SFCM); (l) GM (HAFSA); (m) CSF (standard segmentation); (n) CSF (FCM); (o) CSF (SFCM); (p) CSF (HAFSA).

The second experiment is on MRI, introducing MRI with 5% salt and pepper noise to verify the segmentation effectiveness of HAFSA in the real world. MRI includes 4 parts: the white matter (WM), gray matter (GM), cerebrospinal fluid (CSF), and background. Hence, MRI can be divided into 4 clusters [10]. The first column of Figure 5 presents the original MRI, standard segmentation of WM, standard segmentation of GM, and standard segmentation of CSF; from the second column to the fourth column are the segmentation results of FCM, SFCM, and HAFSA, respectively. Observing the first row of Figure 5, the boundaries segmented by FCM and SFCM are fuzzy but HAFSA is much clearer. For further comparison, the segmentation results of 3 algorithms have been divided into 3 target regions which are the white matter (WM), the gray matter (GM), and the cerebrospinal fluid (CSF), as shown in the second to the fourth rows of Figure 5. The basic FCM algorithm and the SFCM algorithm obtain the inferior effectiveness of segmentation whose edges are in the 3 target regions with a lot of trivial and rough areas, and the details of the target regions cannot be distinguished clearly. The proposed HAFSA can restore the original details even under complicated noise environment. The segmentation figures generated by HAFSA usually have clear outline, complete targets, and the maximum similarity with the standard segmentations which are divided manually by experienced experts.

Table 2 presents 7 evaluation indexes of 3 algorithms on MRI. The evaluation indexes *J*, PSNR, vpc, vpe, Accuracy, JS, and MSE of HAFSA are superior to the ones of FCM and SFCM obviously; only vpe is inferior to SFCM slightly. Table 2 shows that HAFSA has precise segmentation results on MRI, which means that HAFSA is suitable for practical application.

The CPU times of FCM, SFCM, and HAFSA are 1.6821 seconds, 3.9431 seconds, and 4.4052 seconds on the artificial grid graph and 1.7890 seconds, 4.2350 seconds, and 4.6943 seconds on MRI, respectively. HAFSA is slower than FCM and almost as fast as SFCM.

## 5. Conclusion

The proposed algorithm, HAFSA, is a combination of AFSA, noise reduction mechanism, and Metropolis criterion, and it has the advantages of insensitiveness to initial values, powerful capability of antinoise, robust and precise segmentation result, and fast convergence. The experimental results which are verified by the visual effects and evaluation indexes, *J*, PSNR, vpc, vpe, Accuracy, JS, and MSE, demonstrate that HAFSA is superior to classical FCM and SFCM on both artificial grid graph and actual MRI.

## Figures and Tables

**Algorithm 1:**
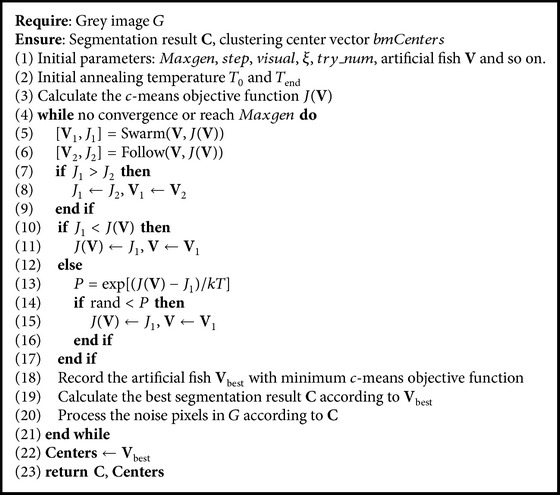
HAFSA.

**Table 1:** Simulation results of grid graph.

Simulation image	Algorithm	Evaluation indexes						
		*J*	PSNR	vpc	vpe	Accuracy	JS	MSE
Grid graph	All 3 (≈)	**0**	**55.4126**	**1**	**0**	**1**	**1**	**0**

5% Gaussian	FCM	1.86*E* + 07	49.4164	0.7674	0.4659	0.9905	0.9813	1.8768
	SFCM	1.86*E* + 07	49.1265	0.7606	0.4763	0.9912	0.9825	1.8259
	HAFSA	3.71**E** + 06	**55.4126**	**0.8041**	** 0.3950**	**0.9997**	**0.9994**	**0.0661**

10% Gaussian	FCM	2.11*E* + 07	38.5015	0.6907	0.5757	0.9874	0.9752	5.4161
	SFCM	2.12*E* + 07	38.5015	0.6858	0.5824	0.9877	0.9756	5.3893
	HAFSA	4.22**E** + 06	**55.4126**	**0.7026**	** 0.5396**	**0.9992**	**0.9983**	**0.1984**

**Table 2:** Simulation results of MRI.

Simulation image	Algorithm	Evaluation indexes						
		*J*	PSNR	vpc	vpe	Accuracy	JS	MSE
MRI	FCM	1.23*E* + 07	17.9176	0.7735	0.6043	0.6554	0.4874	45.2549
	SFCM	1.24*E* + 07	17.9176	0.8052	**0.5860**	0.6890	0.5255	44.2323
	HAFSA	9.07**E** + 06	**20.7944**	**0.8129**	0.5876	**0.9435**	**0.8930**	**32.9370**
